# Impact of Air Pollutants on Outpatient Visits for Acute Respiratory Outcomes

**DOI:** 10.3390/ijerph14010047

**Published:** 2017-01-05

**Authors:** Ran Li, Ning Jiang, Qichen Liu, Jing Huang, Xinbiao Guo, Fan Liu, Zhancheng Gao

**Affiliations:** 1 Department of Respiratory and Critical Care Medicine, Peking University People’s Hospital, Beijing 100044, China; liran2004@163.com (R.L.); aleckdonj@gmail.com (N.J.); liufan@pkuph.edu.cn (F.L.); 2 School of Public Health, Peking University, Beijing 100871, China; liuqichen@bjmu.edu.cn (Q.L.); huangjing2225@163.com (J.H.); guoxb@bjmu.edu.cn (X.G.)

**Keywords:** air pollutant, respiratory outcome, outpatient visit

## Abstract

The air pollution in China is a severe problem. The aim of our study was to investigate the impact of air pollutants on acute respiratory outcomes in outpatients. Outpatient data from 2 December 2013 to 1 December 2014 were collected, as well as air pollutant data including ozone (O_3_), nitrogen dioxide (NO_2_), carbon monoxide (CO), sulfur dioxide (SO_2_), and particulate matter (PM_2.5_ and PM_10_). We screened six categories of acute respiratory outcomes and analyzed their associations with different air pollutant exposures, including upper respiratory tract infection (URTI), acute bronchitis (AB), community-acquired pneumonia (CAP), acute exacerbation of chronic obstructive pulmonary disease (AECOPD), acute exacerbation of asthma (AE-asthma), and acute exacerbation of bronchiectasis (AEBX). A case-crossover design with a bidirectional control sampling approach was used for statistical analysis. A total of 57,144 patients were enrolled for analysis. PM_2.5_, PM_10_, NO_2_, SO_2_, and CO exposures were positively associated with outpatient visits for URTI, AB, CAP, and AEBX. PM_10_, SO_2_, and CO exposures were positively associated with outpatient visits for AECOPD. Exposure to O_3_ was positively associated with outpatient visits for AE-asthma, but negatively associated with outpatient visits for URTI, CAP, and AEBX. In conclusion, air pollutants had acute effects on outpatient visits for acute respiratory outcomes, with specific outcomes associated with specific pollutants.

## 1. Introduction

Air pollution has become an increasing concern in China, with Beijing being located in the area most seriously affected by haze [1]. Air pollutants are proven to have multiple adverse influences on human health, with the respiratory and cardiovascular systems being most commonly involved. Air pollutants have both acute and chronic effects on the respiratory system. Acute respiratory outcomes include two forms: acute respiratory diseases, and acute exacerbations of chronic respiratory diseases. Previous studies have mainly focused on the effects of air pollutants on hospitalization, emergency visits, and mortality associated with multiple acute respiratory outcomes. These studies are largely based on correlations of epidemiological and environmental data. Ozone (O_3_), nitrogen dioxide (NO_2_), carbon monoxide (CO), sulfur dioxide (SO_2_), and particulate matter (PM_10_ and PM_2.5_) are the main ambient pollutants. The literature shows that exposure to air pollutants increases the risk of hospitalization, emergency visits and mortality. O_3_, NO_2_, CO, SO_2_, PM_10_, and PM_2.5_ are all proven to increase hospitalization rates with acute exacerbation of chronic obstructive pulmonary disease (AECOPD) or respiratory infections [2,3,4,5,6,7,8]. NO and PM_2.5_ exposures are associated with hospitalization for community-acquired pneumonia (CAP) [9]. Air pollutant exposure also increases asthma admissions [8,10]. Increased concentrations of PM, NO_2_, and SO_2_ have been shown to increase the risk of emergency room visits for upper respiratory tract infection (URTI), pneumonia, and chronic obstructive pulmonary disease (COPD) [11]. Numerous studies have shown that exposure to multiple air pollutants is associated with emergency room visits for asthma [10,11,12]. PM_10_, O_3_, and NO_2_ are associated with mortality from respiratory diseases in both developed and developing countries [2,3,13,14]. However, some studies have reported that exposure to air pollutants has little effect on hospitalization for respiratory diseases [15], while other studies have reported that ambient CO exposure is associated with a decrease in hospitalizations for AECOPD and respiratory tract infections [16,17].

The impact of air pollutants on outpatient visits for acute respiratory outcomes has rarely been reported. A study in Hong Kong found air pollutants could increase the general outpatient clinic consultations for upper respiratory tract infections [18]. Another study showed that NO_2_ and particulate matter exposures were associated with increased doctor visits for acute symptomatic episodes of COPD [19]. Nevertheless, evidence of the relationship between air pollutants and outpatient visits for acute respiratory outcomes is lacking. Here we analyzed the association between air pollutants and outpatient visits for acute respiratory outcomes from 2 December 2013 to 1 December 2014 in Peking University People’s Hospital, Beijing, China.

## 2. Materials and Methods 

### 2.1. Outpatient Data

Respiratory outpatient department and fever clinic are important locations of initial visits for patients with acute respiratory diseases or acute exacerbation of chronic respiratory diseases in China. Hospitalization data has some limitations as a measure of the acute effect of air pollutants in China since patients often wait several days for a hospital bed. Using emergency visits to estimate the acute effect is also problematic. The medical care system in China differs from developed countries, with outpatient services being equally convenient as emergency departments. When acute illness episodes arise, patients are able to attend outpatients immediately, without making an appointment in advance. Patients with mild or moderate disease usually expect to receive diagnosis and treatment from respiratory specialists in outpatient department, rather than from physicians in emergency departments, who are not respiratory specialists. As a result, patients with acute episodes of diseases often prefer to visit specialized outpatient departments rather than the emergency department. Moreover, febrile patients of all causes are required first to visit a fever clinic before transferring to other departments, resulting in a large number of patients with acute respiratory outcomes being diagnosed and treated in fever clinics. If the condition of a patient is assessed to be severe by clinicians in the respiratory outpatient department or fever clinics, patients are transferred to the emergency department or directly to the ward. Hence respiratory outpatient departments and fever clinics, rather than emergency departments, receive the vast majority of patients with acute respiratory outcomes. Therefore in China, outpatient visits are a better reflection of the acute effect of air pollutants than hospitalizations and emergency visits, and analyzing outpatient data permits a more comprehensive estimate of the impact of air pollutant exposures, especially for mild and moderate episodes.

Outpatient data were collected from the respiratory department and the fever clinic in the outpatient database of Peking University People’s Hospital. Collected information included visit date, sex, age, medical history, and diagnosis. Patients were screened for acute respiratory outcomes, which were classified into six mutually exclusive categories, including URTI, acute bronchitis (AB), CAP, AECOPD, acute exacerbation of asthma (AE-asthma), and acute exacerbation of bronchiectasis (AEBX). URTI was defined as acute inflammation induced by viruses or bacteria which was confined to the upper respiratory tract, which included the diagnosis of common cold, pharyngitis, laryngitis, and tonsillitis. AB was defined as acute inflammation of the trachea and bronchi, presenting with cough with or without sputum, and without pulmonary infiltrates in chest imaging. If chest imaging showed infiltrates of the lung which were considered due to infections, excluding tuberculosis and fungal infections, CAP was diagnosed regardless of whether there were underlying chronic respiratory diseases or not. AECOPD, AE-asthma and AEBX were defined as an acute worsening of respiratory symptoms exceeding daily variation in patients with a previous diagnosis of COPD, asthma, and bronchiectasis, respectively, without confirmed pneumonia in chest imaging. Overlapping COPD, asthma or bronchiectasis were excluded. Outpatient visits for chronic diseases without an acute exacerbation, non-infectious diseases, and diseases without a definite diagnosis were excluded. Duplicated visits in the same day were also excluded. The clinical diagnosis was made by outpatient physicians and was reviewed by study researchers. The need for informed consent was waived since the study was retrospective and all data were anonymized prior to analysis. The study was conducted in accordance with the Declaration of Helsinki, and the protocol was approved by the Ethics Committee of Peking University People’s Hospital (Project identification code: 2014PHB120-01).

### 2.2. Environmental Data

The population of Beijing is quite mobile and large hospitals such as Peking University People’s Hospital receive patients from various areas of the city. Therefore, average values of daily environmental data from 12 monitoring stations throughout the city were adopted to estimate the exposure of air pollutants, including eight urban stations, three suburban stations, and one background station. Daily mean concentrations of six main air pollutants were collected from the surveillance data of the China Meteorological Administration, including O_3_, NO_2_, CO, SO_2_, PM_10_, and PM_2.5_. The daily concentrations of air pollutants were calculated as the 24 h mean concentration, except for O_3_, which was calculated as the 8 h mobile mean concentration. The unit of the air pollutants was μg/m^3^, except for CO for which the unit was mg/m^3^, as adopted in the Chinese air quality index (AQI). The individual air quality index (IAQI) of each pollutant was calculated, and AQI was defined as the maximum IAQI, in order to determine the main pollutant. Information on daily mean air pressure (PRS-mean), daily mean air temperature (T-mean), daily maximum air temperature (T-max), and daily mean relative humidity (RH-mean) were also collected for controlling as potential confounding factors.

### 2.3. Statistical Analysis

We fitted a case-crossover design for statistical analysis, which was first proposed by Maclure in 1991 [20], with a bidirectional control sampling approach of 1:3. Cases were defined as the days when an outpatient visit occurred. Retrospective controls were the seventh, 14th, and 21st days without visits prior to the outpatient visit. Prospective controls were the seventh, 14th, and 21st days without visits following the outpatient visit. The integer week was selected as the interval for controlling the effect of day of the week. The days for comparison were close to each other in order to control for the effect of seasonal trends. Since the impact of air pollutants on acute respiratory outcomes may have lag effects, six-day lags were explored from zero to five days after visits. Seven-day holiday periods including the Spring Festival and National Day of China were excluded as these periods are associated with a reduced rate of outpatient visits. Spearman rank correlation analysis was used to identify the correlation of different air pollutants. Meteorological factors were taken into the model as the independent variables, and multiple regression analysis was used to control for the meteorological factors. A conditional logistic regression was applied to study the association between acute respiratory outcomes and air pollutants. The odds ratios were estimated with each 1 μg/m^3^ increment of NO_2_, SO_2_, CO, PM_10_, and PM_2.5_, and with each 1 mg/m^3^ increment of O_3_. The analysis was conducted using SAS software (Statistical Analysis System, version 9.1, SAS Institute Inc., Cary, NC, USA).

## 3. Results

**Figure 1:**
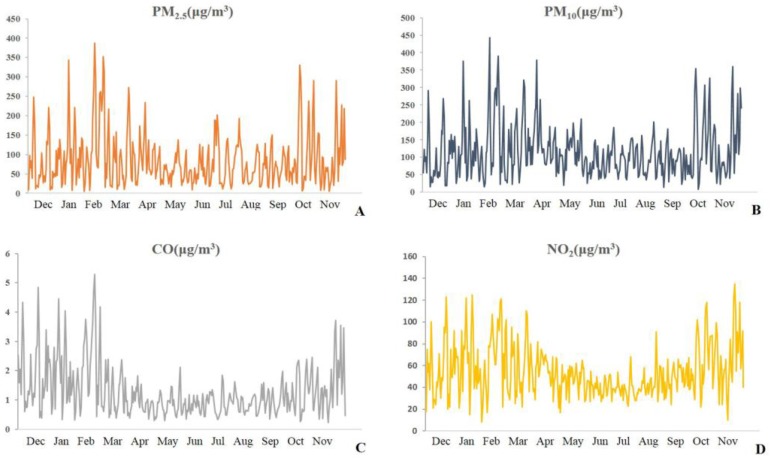

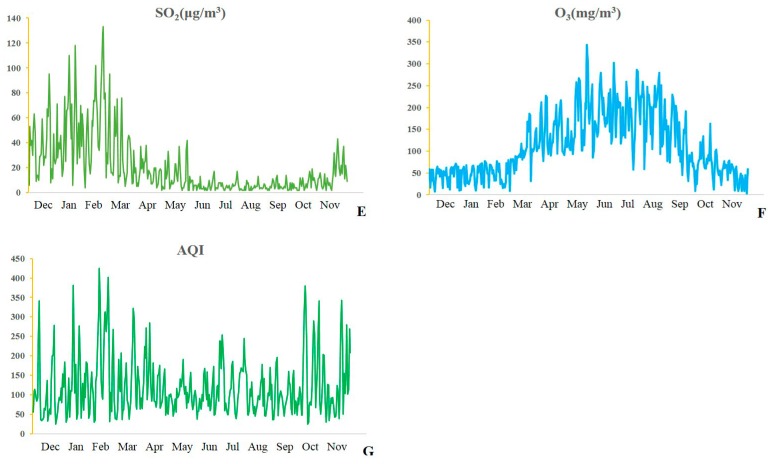
Variation of different air pollutants throughout the study year in panels **A**–**F**. (**A**) Variation of PM_2.5_ in the study year; (**B**) Variation of PM_10_ in the study year; (**C**) Variation of CO in the study year; (**D**) Variation of NO_2_ in the study year; (**E**) Variation of SO_2_ in the study year; (**F**) Variation of O_3_ in the study year; (**G**) Variation of AQI in the study year. Maximal concentrations of air pollutants occurred during cold periods, except for O_3_, which showed an inverse trend. Panel **G** shows the variation of the Chinese AQI, which is a composite measure of air pollutants.

A total of 122,586 visits to the respiratory department and the fever clinic between 2 December 2013 and 1 December 2014 were screened. After screening, a total of 57,144 patients with acute respiratory outcomes were included for analysis, including 36,615 cases of URTI, 10,868 cases of AB, 7015 cases of CAP, 1015 cases of AECOPD, 459 cases of AE-asthma, and 1172 cases of AEBX. The male-to-female ratio was 0.78. The median daily values of the pollutants, the meteorological parameters, and the outcomes during the study period are shown in Table 1. The median daily visits during the study year were 154 for total acute respiratory outcomes. The daily variation of different pollutants and Chinese AQI throughout the study year are shown in Figure 1. Concentrations of PM_2.5_, PM_10_, CO, and SO_2_ were maximal in February. The maximum concentrations of NO_2_ and O_3_ were in October and June, respectively. The maximum value of the AQI, which integrates the effect of different pollutants, was in February. PM was the dominant pollutant during cold periods, while O_3_ was the dominant pollutant during warm periods. Positive correlations were found between NO_2_, CO, SO_2_, PM_10_, and PM_2.5_ (Table 2). However, O_3_ showed a negative correlation with NO_2_, CO, and SO_2_. Since O_3_ was closely related to other pollutants as a secondary pollutant, a single pollutant model was used to analyze the association between air pollutants and acute respiratory outcomes.

**Figure 2:**
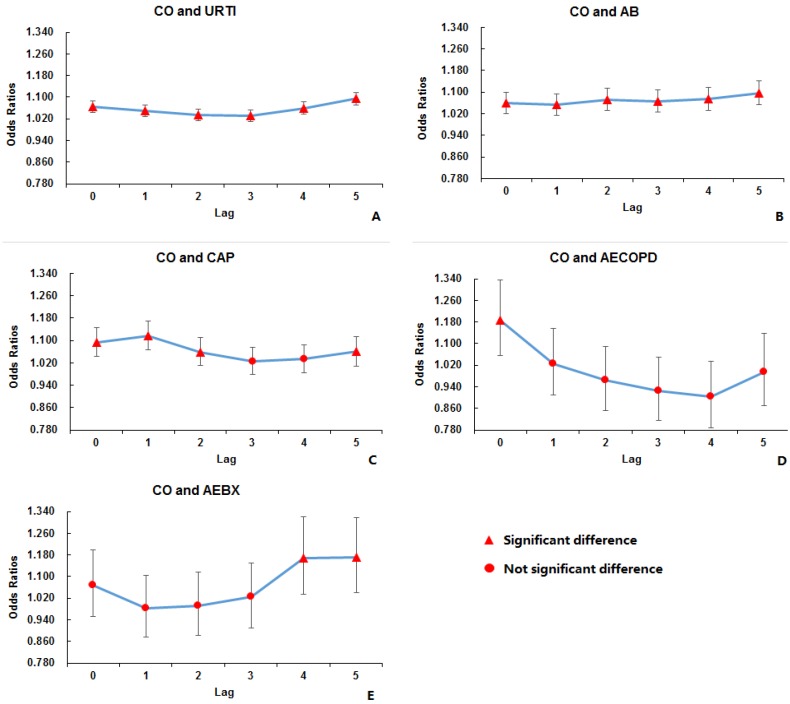
Association between CO and outpatient visits for different acute respiratory outcomes in panels **A**–**E**. (**A**) Association between CO and outpatient visits for URTI; (**B**) Association between CO and outpatient visits for AB; (**C**) Association between CO and outpatient visits for CAP; (**D**) Association between CO and outpatient visits for AECOPD; (**E**) Association between CO and outpatient visits for AEBX. Horizontal axis represents the days lagged from 0–5, and vertical axis represented the odds ratios. The highest odds ratios appeared in the association with AECOPD in lag 0 (odd ratio (OR) = 1.188, 95% confidence interval (CI) 1.056–1.335).

After adjusting for air pressure, temperature, relative humidity, and holidays, the associations between air pollutants and acute respiratory outcomes with different lag effects are shown in Table 3. PM_2.5_, PM_10_, NO_2_, SO_2_, and CO had a positive association with outpatient visits for URTI, AB, CAP, and AEBX. PM_10_, SO_2_, and CO had a positive association with outpatient visits for AECOPD. O_3_ had a weak negative association with outpatient visits for URTI, CAP, and AEBX. However, O_3_ was the sole pollutant which had a positive association with outpatient visits for AE-asthma in lag 4 (odd ratio (OR) = 1.004, 95% confidence interval (CI) 1.001–1.007). For lag effects, all the associated air pollutants showed immediate effects on outpatient visits for URTI, AB, and AECOPD (lag 0). The effects on visits for CAP were also immediate, except for PM_10_, which showed a one-day lag. The effect on visits for AEBX showed four- to five-day lags for all the associated pollutants. It was not possible to estimate the maximal lag effects for all the air pollutants, while CO showed the most obvious trends of lag effects on different lagged days (Figure 2).

## 4. Discussion

Our study found that air pollutants were positively associated with outpatient visits for different acute respiratory outcomes, demonstrating that air pollutants increased the risk for multiple respiratory diseases. The mechanism of the impact of air pollutants on respiratory illnesses is not fully understood, and possible mechanisms include destroying the airway epithelial barrier, interfering with cellular signaling pathways, destroying the lung parenchyma, various forms of inflammation response, impairing cell immunity, epigenetic modifications, and autophagy [21], while inflammation is likely to be a central mechanism. Pulmonary inflammation is considered to be produced via two pathways: one is oxidative stress induced by free oxygen radicals, which can be produced from pollutants, and the other is decreased immunologic function as a result of the suppressed function of macrophages. Studies in vitro and in vivo have demonstrated the effect of air pollutants on the respiratory system, with most work focusing on PM. Different-sized fractions of ambient PM can induce inflammatory and toxic responses in alveolar cells, increasing the release of the proinflammatory cytokines IL-8 and IL-6 [22]. Increased IL-8 expression in bronchial epithelial cells can also be induced by PM through the activation of the epidermal growth factor receptor (EGFR) signaling pathway [23]. In vivo, PM_2.5_ exacerbated an ongoing streptococcal infection in rats; transition metals such as iron and nickel in PM_2.5_ reduced pulmonary bacterial clearance in infected rats; and iron showed immunotoxicity, even in uninfected rats [24]. The metal-mediated lung injury was enhanced in rat models with fundamental cardiopulmonary diseases [25]. Moreover, PM_2.5_ can induce airway hyper-responsiveness with the synergistic effects of insoluble and soluble components [26]. Further studies on the mechanism are needed in order to fully understand the impact of air pollutants on multiple respiratory outcomes.

The case-crossover design has been widely adopted to estimate the acute effect of exposures. We chose a bidirectional design, since severe analytical bias may occur for exposures with time trends if a unidirectional design is used [27]. The seasonal trend is the dominant variation factor during a single year which can be controlled well by a bidirectional design. Our analysis showed positive correlations between different pollutants, and a single pollutant model was adopted to avoid the impact of collinearity.

Higher concentrations of air pollutants during cold seasons, except for O_3_, in Beijing are likely to be the result of pollutants being discharged from heating systems that use biomass or other fuels. More O_3_ is produced in warm seasons due to the effect of stronger ultraviolet light. Our study showed positive associations between air pollutant exposures and acute respiratory outcomes, consistent with previous studies. Different air pollutants had influences at different levels with different lag effects, which is consistent with the heterogeneity shown in other published literature. Different metrics methods may influence the estimates of the impacts of air pollutant exposures. In our study, the metric of exposure is 1 μg/m^3^ for the air pollutants, except for CO with the metric of 1 mg/m^3^, in contrast with the 10 μg/m^3^ or an increase equal to the interquartile range of the pollutant distribution adopted in other literature. Beijing is a severely polluted city with a large population of over 20 million: we identified over 100 outpatient visits for acute respiratory outcomes in one day in one hospital alone. Across the whole city the population affected is very large, which could result in significant social and economic burdens. Our study covered six main air pollutants and six main acute respiratory outcomes. This makes our study one of the most comprehensive analyses reported in the literature.

PM is the factor most widely studied as a contributor to the burden of acute respiratory disorders. Nevertheless, the main gaseous pollutants (CO, NO_2_, and SO_2_) also play an important role, reminding us that pollutants are a crucial consideration. CO is usually considered as primarily an indoor pollutant. However, ambient CO is mainly derived from indoor sources, especially from heating systems during the winter. A negative association between O_3_ and several acute respiratory outcomes has been previously reported in a study which showed a protective association between O_3_ and pneumonia [5]. In addition, the negative associations were weak in our study, and were in accordance with the inverse seasonal trend compared with other pollutants.

Our study has some limitations. We chose outpatient data of only one hospital to estimate the impacts of air pollutants. Since different hospitals may have different disease constitutions in outpatients, and the number of outpatient visits in one hospital is relatively small for a whole city, a possible selection bias could affect the result. However, with the high population mobility in Beijing, we think data from one large hospital could at least partially reflect the overall impact of the air pollutants.

## 5. Conclusions 

Our study has shown that air pollutants have an acute effect on a variety of acute respiratory outcomes in outpatients. Gaseous pollutants and PM were equally harmful. Prevention and control of air pollution remains a major challenge in China.

## Figures and Tables

**Table 1:** Median daily values of pollutants, meteorological parameters, and outcomes.*

Pollutants	Median	Meteorological Parameters	Median	Outcomes	Median
PM_2.5_ (μg/m^3^)	66	PRS-mean (kPa)	101.24	URTI (no.)	96
PM_10_ (μg/m^3^)	101	T-mean (°C)	15.5	AB (no.)	28
CO (mg/m^3^)	1.01	T-max (°C)	21.5	CAP (no.)	18
NO_2_ (μg/m^3^)	49	RH-mean (%)	52 ± 19 *	AECOPD (no.)	2
SO_2_ (μg/m^3^)	11			AE-asthma (no.)	1
O_3_ (μg/m^3^)	92			AEBX (no.)	3

* All of the data were not in accordance with a normal distribution during the study period and were described by medians, except for the RH-mean, which was described by the mean ± SD. PRS-mean: daily mean air pressure; T-mean: daily mean air temperature; T-max: daily maximum air temperature; RH-mean: daily mean relative humidity; URTI: upper respiratory tract infection; AB: acute bronchitis; CAP: community-acquired pneumonia; AECOPD: acute exacerbation of chronic obstructive pulmonary disease; AE-asthma: acute exacerbation of asthma; AEBX: acute exacerbation of bronchiectasis; no.: number.

**Table 2:** Correlation coefficients among air pollutants.

Pollutants	PM_2.5_	PM_10_	CO	NO_2_	SO_2_	O_3_
**PM_2.5_**	1.000	0.877 *	0.852 *	0.675 *	0.476 *	−0.073
**PM_10_**	0.877 *	1.000	0.713 *	0.735 *	0.515 *	−0.024
**CO**	0.852 *	0.713 *	1.000	0.748 *	0.684 *	−0.396 *
**NO_2_**	0.675 *	0.735 *	0.748 *	1.000	0.634 *	−0.361 *
**SO_2_**	0.476 *	0.515 *	0.684 *	0.634 *	1.000	−0.474 *
**O_3_**	−0.073	−0.024	−0.396 *	−0.361 *	−0.474 *	1.000

* Significant difference (*p* < 0.05).

**Table 3:** Associations between air pollutants and outpatient visits for acute respiratory outcomes.

Outcomes	PM_2.5_	PM_10_	CO	NO_2_	SO_2_	O_3_
	ORs 95% CI	ORs 95% CI	ORs 95% CI	ORs 95% CI	ORs 95% CI	ORs 95% CI
**URTI**						
lag 0	1.001 * 1.000–1.001	1.001 * 1.000–1.001	1.064 * 1.042–1.086	1.002 * 1.002–1.003	1.003 * 1.002–1.004	1.000 1.000–1.000
lag 1	1.000 1.000–1.001	1.000 1.000–1.001	1.049 * 1.028–1.071	1.002 * 1.001–1.002	1.002 * 1.002–1.003	0.999 * 0.999–1.000
lag 2	1.000 1.000–1.000	1.000 1.000–1.000	1.033 * 1.012–1.055	1.001 * 1.000–1.001	1.001 * 1.001–1.002	0.999 * 0.999–0.999
lag 3	1.000 1.000–1.001	1.000 1.000–1.000	1.032 * 1.010–1.054	1.001 * 1.001–1.002	1.002 * 1.001–1.003	1.000 0.999–1.000
lag 4	1.001 * 1.000–1.001	1.000 1.000–1.001	1.060 * 1.037–1.083	1.002 * 1.001–1.003	1.002 * 1.002–1.003	1.000 1.000–1.001
lag 5	1.001 * 1.001–1.001	1.001 * 1.000–1.001	1.095 * 1.071–1.118	1.003 * 1.002–1.003	1.003 * 1.002–1.004	1.000 1.000–1.001
**AB**						
lag 0	1.001 * 1.000–1.001	1.001 * 1.000–1.001	1.059 * 1.020–1.099	1.003 * 1.001–1.004	1.002 * 1.001–1.004	1.000 0.999–1.000
lag 1	1.001 * 1.000–1.001	1.001 * 1.000–1.001	1.053 * 1.014–1.094	1.001 1.000–1.003	1.002 * 1.001–1.004	1.000 0.999–1.001
lag 2	1.001 * 1.001–1.002	1.001 * 1.000–1.001	1.072 * 1.032–1.114	1.002 * 1.000–1.003	1.003 * 1.001–1.004	1.000 0.999–1.000
lag 3	1.001 * 1.000–1.001	1.001 * 1.000–1.001	1.066 * 1.026–1.107	1.002 * 1.001–1.004	1.004 * 1.002–1.005	1.000 0.999–1.000
lag 4	1.001 * 1.000–1.001	1.001 * 1.000–1.001	1.074 * 1.033–1.118	1.003 * 1.001–1.004	1.003 * 1.002–1.004	1.000 0.999–1.001
lag 5	1.001 * 1.000–1.001	1.001 * 1.000–1.001	1.097 * 1.054–1.141	1.002 * 1.001–1.004	1.003 * 1.001–1.004	1.000 0.999–1.000
**CAP**						
lag 0	1.001 * 1.000–1.001	1.000 1.000–1.001	1.093 * 1.044–1.146	1.002 * 1.000–1.004	1.003 * 1.002–1.005	0.999 * 0.998–1.000
lag 1	1.001 * 1.000–1.001	1.001 * 1.000–1.001	1.117 * 1.066–1.170	1.003 * 1.001–1.004	1.004 * 1.002–1.006	0.999 * 0.998–1.000
lag 2	1.000 1.000–1.001	1.001 * 1.000–1.001	1.059 * 1.010–1.111	1.002 1.000–1.003	1.003 * 1.002–1.005	1.000 0.999–1.000
lag 3	1.000 1.000–1.001	1.000 1.000–1.000	1.026 0.978–1.076	1.001 0.999–1.003	1.003 * 1.001–1.005	1.000 0.999–1.000
lag 4	1.000 1.000–1.001	1.000 1.000–1.001	1.033 0.983–1.085	1.001 0.999–1.003	1.002 * 1.001–1.004	0.999 0.999–1.000
lag 5	1.001 * 1.000–1.001	1.000 1.000–1.001	1.060 1.009–1.113	1.001 1.000–1.003	1.003 * 1.001–1.004	1.000 0.999–1.001
**AECOPD**						
lag 0	1.001 1.000–1.003	1.001 * 1.000–1.003	1.188 * 1.056–1.335	1.004 1.000–1.008	1.005 * 1.001–1.010	1.002 1.000–1.004
lag 1	1.001 0.999–1.002	1.000 0.999–1.002	1.025 0.910–1.155	1.001 0.997–1.005	1.002 0.997–1.007	1.000 0.998–1.002
lag 2	1.001 0.999–1.002	1.000 0.998–1.001	0.963 0.851–1.090	0.999 0.994–1.003	1.000 0.995–1.005	1.000 0.998–1.002
lag 3	1.000 0.998–1.001	0.999 * 0.997–1.000	0.924 0.814–1.049	0.998 0.994–1.003	0.998 0.993–1.002	0.999 0.997–1.001
lag 4	0.999 0.998–1.001	1.000 0.998–1.001	0.903 0.789–1.033	0.998 0.994–1.003	0.998 0.994–1.003	0.999 0.998–1.001
lag 5	1.000 0.998–1.001	1.000 0.999–1.001	0.996 0.871–1.139	1.000 0.996–1.004	1.000 0.995–1.005	1.000 0.998–1.002
**AE-asthma**						
lag 0	1.001 0.999–1.003	1.000 0.998–1.002	1.013 0.848–1.209	1.000 0.994–1.006	1.000 0.993–1.006	1.000 0.997–1.003
lag 1	1.001 0.999–1.003	1.000 0.999–1.002	1.147 0.956–1.375	1.005 0.999–1.011	1.004 0.997–1.010	1.000 0.997–1.003
lag 2	0.999 0.997–1.001	0.999 0.997–1.001	0.944 0.790–1.128	0.998 0.991–1.004	0.998 0.991–1.005	1.001 0.998–1.004
lag 3	0.998 0.995–1.000	0.999 0.997–1.001	0.872 0.725–1.047	0.996 0.990–1.003	1.000 0.993–1.006	1.001 0.999–1.004
lag 4	0.999 0.996–1.001	0.999 0.997–1.001	0.899 0.742–1.089	0.994 0.988–1.001	0.999 0.993–1.006	1.004 *1.001–1.007
lag 5	1.000 0.998–1.002	1.0000.998–1.002	1.047 0.868–1.261	1.001 0.995–1.007	1.001 0.995–1.008	1.001 0.998–1.004
**AEBX**						
lag 0	1.000 0.998–1.001	1.000 0.999–1.001	1.069 0.952–1.199	1.000 0.996–1.004	1.002 0.997–1.006	0.999 0.997–1.000
lag 1	0.999 0.998–1.000	1.000 0.999–1.001	0.983 0.876–1.103	0.998 0.994–1.002	1.000 0.996–1.004	0.998 * 0.996–1.000
lag 2	0.999 0.998–1.000	0.999 0.998–1.000	0.993 0.881–1.118	0.998 0.994–1.002	1.000 0.995–1.004	0.999 0.997–1.000
lag 3	1.000 0.998–1.001	0.999 0.998–1.001	1.024 0.911–1.150	0.999 0.995–1.003	1.000 0.996–1.004	0.998 * 0.996–1.000
lag 4	1.001 1.000–1.003	1.001 1.000–1.002	1.169 * 1.035–1.320	1.006 * 1.002–1.010	1.005 * 1.001–1.010	0.999 0.997–1.001
lag 5	1.001 * 1.000–1.003	1.001 * 1.000–1.003	1.170 * 1.039–1.318	1.003 0.999–1.007	1.004 0.999–1.008	1.000 0.999–1.002

* Significant difference (*p* ˂ 0.05).
